# A Novel Nitrite-Base Aerobic Denitrifying Bacterium *Acinetobacter* sp. YT03 and Its Transcriptome Analysis

**DOI:** 10.3389/fmicb.2019.02580

**Published:** 2019-11-15

**Authors:** Bin Li, Ran Lv, Ying Xiao, Wei Hu, Yuliang Mai, Jingwen Zhang, Lan Lin, Xiaoyong Hu

**Affiliations:** Guangdong Provincial Key Laboratory of Industrial Surfactant, Guangdong Research Institute of Petrochemical and Fine Chemical Engineering, Guangzhou, China

**Keywords:** aerobic denitrification, nitrite removal, *Acinetobacter* sp., transcriptome analysis, differentially expressed gene

## Abstract

Nitrite in a water environment is very harmful to humans and aquatic animals. A novel aerobic denitrifying bacterium able to utilize ${\text{NO}}_{2}^{-}$-N as the only nitrogen source was isolated for the purpose of removing nitrite from water, which was identified as *Acinetobacter* sp. and named as YT03. The growth and denitrification activity of strain YT03 was assessed comprehensively. Results showed that the nitrite in water with an initial concentration of 10 mg L^–1^ could be completely removed within 6 h by strain YT03, and the optimal conditions for strain YT03 to remove nitrite were as follows: sodium succinate as the carbon source, C/N ratio of 16, pH of 6.5, temperature of 30°C, and shaking speed of 250 rpm. An RNA-Seq transcriptome analysis was used to find genes associated with nitrite removal. Compared with the removal of ammonia nitrogen, 47 genes were significantly differentially expressed, including 20 up-regulated and 27 down-regulated genes, mainly involved in the transport process, biosynthetic process, and so on. And among the differentially expressed genes, C4-dicarboxylate transporter (*Dct*A) and nitrate/nitrite transporter (*Nrt*) might be of importance for the efficient utilization of carbon and nitrogen sources in aerobic nitrite denitrification with sodium succinate by strain YT03.

## Introduction

Nitrite pollution has become a considerable problem in water environments and is generally caused by the insufficient disposal of industrial and domestic wastewater, the excessive uses of nitrogen fertilizers, and also natural nitrogen cycle including the metabolism of aquatic organisms (Alonso and Camargo, 2006; Yilmaz et al., 2006; Song et al., 2011; He et al., 2017; Hui et al., 2018). The nitrite issue is particularly serious in the aquaculture industry. The application of the intensive culture system improves the aquatic products’ yields, however, at the same time, excess nitrite was easily accumulated by uneaten high protein feed and feces under nitrogen cycle in such recirculating aquaculture system (Barman et al., 2017). Nitrite poses a severe threat to aquatic animals, and further to humans, by triggering a series of negative physiological effects, resulting in illness or even death (Tseng and Chen, 2004; He et al., 2017). Therefore, it is very important to control the concentration of nitrite in aquaculture water and other nitrite polluted waters.

Biological nitrogen removal method is commonly adopted for the elimination of nitrite pollution since it is much more efficient and economical than physical and chemical techniques (United States Environmental Protection Agency [USEPA], 1993; Ahn, 2006; Khardenavis et al., 2007; do Canto et al., 2008; Wang et al., 2013). By means of biological method, nitrite could be nitrified to nitrate by some nitrite-oxidizing bacteria; however, the resulting nitrate might accumulate and lead to the eutrophication of water, and is very likely to be reduced to hazardous nitrite in human body (Philips et al., 2002; Alawi et al., 2007; Ma et al., 2014; He et al., 2017). Besides, nitrite could be reduced to gaseous products by denitrifying bacteria and released from water environment. Nevertheless, conventional denitrifying bacteria could only grow and utilize nitrite under anoxic or anaerobic conditions. They are usually sensitive to oxygen, and the expression of enzymes related to the denitrification process might be suppressed under aerobic conditions (Zheng et al., 2012; Zhou et al., 2014; Zhao et al., 2018). Aerobic denitrifying bacterium is able to utilize oxygen and nitrate or nitrite as electron acceptors simultaneously under aerobic condition (Yang et al., 2013; Huang et al., 2015; Zhao et al., 2018). Thus, compared to conventional denitrifying bacterium, aerobic denitrifying bacterium might be more suitable for nitrite pollution elimination in scenarios including intensive aquaculture industry where adequate dissolved oxygen was needed for aquatic animals (Li et al., 2008).

Since the existence of aerobic denitrification was confirmed in *Paracoccus denitrificans* (formerly *Thiosphaera pantotropha*) (Robertson and Kuenen, 1984), numerous aerobic denitrifying bacteria belonging to species of *Alcaligenes* (Vanniel et al., 1992; Matsuzaka et al., 2003), *Diaphorobacter* (Khardenavis et al., 2007), *Pseudomonas* (Wan et al., 2011; Zhang et al., 2011; Qiu et al., 2012; Zheng et al., 2014; Zhou et al., 2014; Huang et al., 2015; Zhao et al., 2018), *Acinetobacter* (Ren et al., 2014; Liu et al., 2015; Yang et al., 2016), *Bacillus* (Zhang et al., 2010; Song et al., 2011; Barman et al., 2017), *Marinobacter* (Zheng et al., 2012), and *Paracoccus* (Shi et al., 2013) have been isolated. Nevertheless, among these reports about aerobic denitrifiers, only a few are focused on utilizing nitrite as the only nitrogen source and removing nitrite of low concentrations, especially in aquaculture water, thus it is very urgent to explore more aerobic denitrifying bacteria that utilize and remove low-concentration nitrite from water with high efficiency.

In this study, a novel aerobic denitrifying bacterium was isolated and proved to be highly efficient in nitrite removal; hence, this new aerobic denitrifying bacterium could be very promising for nitrite removal from water environments. Furthermore, high-throughput mRNA sequencing (RNA-Seq), which could simultaneously detect the expression of a large number of genes, was used to reveal the genes closely related to nitrite-base aerobic denitrification.

## Materials and Methods

### Isolation and Identification of Nitrite-Based Aerobic Denitrifying Bacterium

#### Media

The selective medium plate (per liter) consisting of 100 mL nitrite nitrogen solution (1 g L^–1^ of nitrite nitrogen solution prepared by sodium nitrite and distilled water), 5.40 g sodium succinate, 50 mL trace element solution, 1 mL of bromothymol blue solution (1% in ethanol), and 18 g agar was prepared for the screening of aerobic denitrification bacteria. The ingredients of bacterial enrichment medium in per liter distilled water were as follows: 60 mL nitrite nitrogen solution, 3.24 g sodium succinate, and 50 mL trace element solution. The trace element solution contained 6.50 g K_2_HPO_4_⋅3H_2_O, 2.50 g NaCl, 1.22 g MgSO_4_, 0.20 g CaCl_2_, 0.05 g FeSO_4_⋅7H_2_O, and 0.04 g MnSO_4_⋅H_2_O in per liter distilled water.

The basic nitrite denitrification medium for shaking culture experiments contained 10 mL nitrite nitrogen solution, 0.54 g sodium succinate, and 50 mL trace element solution mentioned above in per liter distilled water. Carbon source, C/N ratio, salinity, pH, and cultivation temperature were adjusted separately in order to investigate the effects of different factors on the growth of the bacterium and the degradation of nitrite by the studied bacterium.

The ingredients of medium for ammonium utilization by strain YT03 were similar to the basic nitrite denitrification medium, except that the nitrite nitrogen solution was replaced by ammonium nitrogen solution (1 g L^–1^ of ammonium nitrogen solution prepared by ammonium chloride and distilled water) with same volume in per liter medium.

All the chemicals used in this study were purchased from Guangzhou chemical reagent factory (Guangzhou, China) and all of the medium above were prepared from laboratory distilled water. The pH of the medium was adjusted to 7.2–7.3, and the prepared medium was autoclaved at 120°C for 20 min before used.

#### Isolation of Bacterium

The activated sludge was collected from a wastewater treatment plant in Puning of China. The sludge sample (10 mL) was transferred to a sterile 0.9% NaCl solution (90 mL) in a 250-mL Erlenmeyer flask and shaken at 150 rpm to obtain a homogeneous suspension. Gradient dilutions were then performed, and the resultant bacterial suspensions were spread onto the selective medium plate and incubated at 30°C for 48 h. The resultant blue colonies and colonies with blue haloes were isolated and inoculated to 100 mL bacterial enrichment medium in 250 mL conical flasks and cultured at 150 rpm and 30°C for 18 h to detect their ability of nitrite removal, respectively. The strain with the highest nitrite removal efficiency was suspended in 20% glycerol solution and stored at −80 or −20°C.

#### Phylogenetic Analysis

The genomic DNA of the bacterium was extracted with an EZ-10 Spin Column genomic DNA isolation kit (Sangon, China) following the manufacturer’s instructions. The 16S rRNA gene was amplified by polymerase chain reaction (PCR) using bacterial universal primers F27 (5′-AGAGTTTGATCATGGCTCAG-3′) and R1492 (5′-TACGGTTACCTTGTTACGACTT-3′), and sequenced by Sangon Biocompany (Shanghai, China). The 16S rDNA sequence was compared to those of other bacteria in the GenBank by the BLAST program. A phylogenetic tree was constructed in the MEGA 7.0 program using the neighbor-joining method.

#### Cell Growth and Nitrite Denitrification

To investigate the characteristics of cell growth and nitrite utilization of the isolated strain, 5 mL suspension of pre-incubated stored cells (30°C, 150 rpm, 18 h) was inoculated into 250 mL flasks which contained 100 mL basic nitrite denitrification medium for shaking culture experiments (mentioned in the section “Media”). Flasks with gas-permeable seals were then cultivated aerobically at temperature of 30°C and a shaking speed of 150 rpm for 6 h. The samples were collected from the flasks at 2 h interval to determine the optical density at 600 nm (OD_600_) and concentrations of ammonium nitrogen (${\text{NH}}_{4}^{+}$-N), nitrite nitrogen (${\text{NO}}_{2}^{-}$-N), and nitrate nitrogen (${\text{NO}}_{3}^{-}$-N).

#### Factors Influencing Nitrite Denitrification

A series of shaking culture experiments were carried out to investigate the effects of different factors on nitrite denitrification. The medium was adjusted for different conditions, and the nitrite removal efficiency during the cultivation was inspected to evaluate the effects of these factors. Carbon sources including sodium succinate, sodium acetate, glucose, and sucrose were separately chosen as the sole carbon source in the culture medium with other conditions remaining unchanged. Similarly, different C/N ratios (4, 8, 12, 16, and 20) were provided by altering the amount of carbon source in the medium. Furthermore, the salinity (0, 5, 10, 15, 20, and 25‰), pH (5.5, 6.5, 7.5, 8.5, and 9.5), and temperature (20, 25, 30, 35, and 40°C) were selected as sole variables in the shaking culture experiments with all other conditions controlled as constants.

#### Utilization of Ammonium by Strain YT03

The ability for ammonium nitrogen utilization of the strain YT03 with ammonium chloride as nitrogen source was also tested under similar conditions and procedures in the section “Cell Growth and Nitrite Denitrification,” except that the basic nitrite denitrification medium was replaced by medium for ammonium utilization.

#### Analytical Methods

OD_600_ was measured at 600 nm using a spectrophotometer (Shimadzu UV-2450). Ammonium nitrogen (${\text{NH}}_{4}^{+}$-N) was determined by Nessler’s reagent photometry (APHA, 2012). Nitrite nitrogen (${\text{NO}}_{2}^{-}$-N) was determined by the *N*-(1-naphthalene)-diaminoethane photometry method. Nitrate nitrogen (${\text{NO}}_{3}^{-}$-N) was analyzed by the Thymol spectrophotometry method. The bacterial nitrogen was determined by the standard HACH method for total nitrogen (TN) after the bacterial cell was separated from the sample by centrifugation and diluted in sterile distilled water to the same volume as the initial sample.

### Transcriptome Analysis of Nitrite Degradation by Aerobic Denitrification of Strain YT03

#### Material

Five milliliter suspension of pre-incubated stored cells (30°C, 150 rpm, 18 h) was inoculated into 250 mL flasks which contained 100 mL base medium for shaking culture experiments (mentioned in the Section “Media”). Flasks with gas-permeable seals were then cultivated aerobically at temperature of 30°C and a shaking speed of 150 rpm for 6 h (Marked YT03_Y), using ammonium chloride instead of sodium nitrite in base medium as control group (Marked YT03_A).

#### RNA Extraction, Library Construction, and Sequencing

The total RNA of each of above listed samples was isolated using the Trizol Kit (Promega, United States) following the manufacturer’s instructions. Then the total RNA was treated with RNase-free DNase I (Takara Bio, Japan) for 30 min at 37°C to remove residual DNA. RNA quality was verified using a 2100 Bioanalyzer (Agilent Technologies, Santa Clara, CA, United States) and was also checked by RNase-free agarose gel electrophoresis. The transcriptome assembly library as a reference library was constructed by mixing equal amounts of RNA from the above samples. Next, Poly (A) mRNA was isolated using oligo-dT beads (Qiagen). All mRNA was broken into short fragments by adding fragmentation buffer. First-strand cDNA was generated using random hexamer-primed reverse transcription, followed by the synthesis of the second-strand cDNA using RNase H and DNA polymerase I. The cDNA fragments were purified using a QIAquick PCR extraction kit. These purified fragments were then washed with EB buffer for end preparation poly (A) addition and ligated to sequencing adapters. Following agarose gel electrophoresis and extraction of cDNA from gels, the cDNA fragments were purified and enriched by PCR to construct the final cDNA library. The cDNA library was sequenced on the Illumina sequencing platform (Illumina HiSeq^TM^ 2500) using the paired-end technology by Gene *Denovo* Co. (Guangzhou, China).

#### Differentially Expressed Genes Analysis

Reads Per Kilobase of exon model per Million mapped reads (RPKM) was used to quantitatively estimate gene expression (Mortazavi et al., 2008). Based on Rockhopper, the number of reads of each gene is obtained, and the expression information is obtained by local regression. Then the negative binomial distribution statistical model is used to test the zero hypothesis of each gene expression comparison, and the *P*-value information of gene comparison is obtained. Then the *q*-value of gene comparison is obtained by the Benjamini–Hochberg (BH) multiple modification (Benjamini and Hochberg, 1995). In this study, genes having FDR ≤ 0.05 and FC ≥ 2 were considered as differentially expressed genes (DEGs).

#### Differential Gene Annotation

Using the Gene Ontology (GO^1^) database (Harris et al., 2004), genes can be classified according to the biological processes they participate in, the components that make up cells, and the molecular functions they achieve. The GO annotation statistics of two groups of differentially expressed genes were carried out, and one of the samples was used as the control. The results can be used to draw up and down GO annotation histogram.

#### Differential Gene Enrichment Analysis

The software Goatools^2^ (Klopfenstein et al., 2018) is used for enrichment analysis and Fisher’s exact test is used. In order to control the calculated false positive rate, four multiple tests (Bonferroni, Holm, Sidak, and false discovery rate) were used to correct the *P*-value. Generally, when the corrected *P*-value (p_FDR) is <0.05, it is considered that the GO function is significantly enriched. KEGG pathway enrichment analysis was carried out with KOBAS. The calculation principle was the same as GO function enrichment analysis. Fisher exact test was used for calculation. In order to control the false positive rate, BH (FDR) method was used to conduct multiple tests. Corrected *P*-value threshold was 0.05, KEGG pathway satisfying this condition was defined as KEGG pathway which was significantly enriched in differentially expressed genes.

## Results and Discussion

### Identification of Strain YT03

**FIGURE 1:**
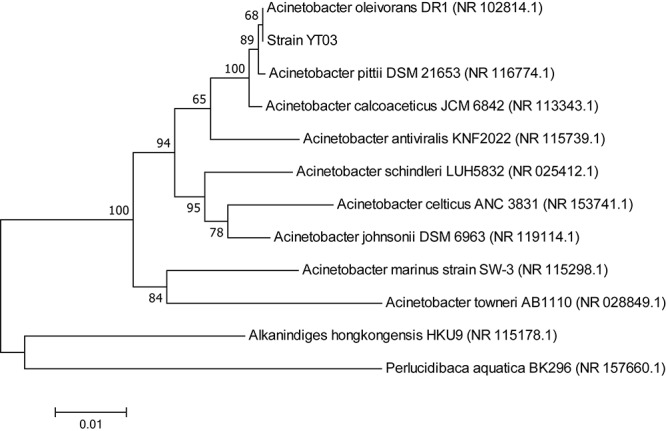
Phylogenetic tree derived from neighbor-joining analysis of partial 16S rRNA gene sequence.

Ten strains isolated from selective medium plates were selected to test their heterotrophic nitrification–aerobic denitrification ability. Among them, strain YT03 showed the strongest and most stable heterotrophic nitrification–aerobic denitrification ability. The partial 16S rRNA sequences of strain YT03 were determined and deposited in the GenBank database (GenBank accession number MN173477). The BLAST results indicated that strain YT03 was closely related to *Acinetobacter oleivorans*. A neighbor-joining phylogenetic tree was constructed based on partial 16S rRNA of the strain YT03 and the closely related bacteria (Figure 1).

### Growth and Nitrite Denitrification by Strain YT03

**FIGURE 2:**
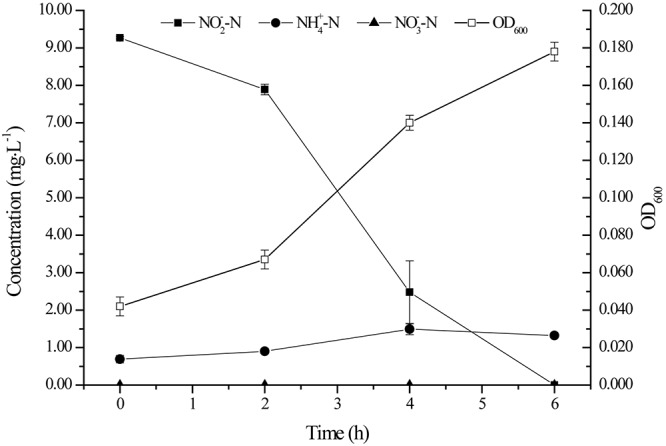
The growth and nitrite removal by strain YT03. Error bars mean ±SD of three replicates.

Figure 2 depicts the growth of strain YT03 and its performance in nitrite removal. Here, OD_600_ was employed as an indicator for the growth of bacterium. In the first 2 h, strain YT03 grew slowly, as it might experience a lag phase. While after that, strain YT03 began to grow rapidly and the OD_600_ value reached 0.178 at last. The ${\text{NO}}_{2}^{-}$-N with an initial concentration of 9.27 mg L^–1^ was removed rapidly, and it could not be detected out after 6 h. During the whole process, the average nitrite denitrification rate of strain YT03 is 1.67 mg L^–1^ h^–1^, and the maximum ${\text{NO}}_{2}^{-}$-N removal rate reached 2.71 mg L^–1^ h^–1^.

During the whole nitrite removal process, nitrate was never detectable, and the slight accumulation of ammonium might be due to the microbial autolysis under famine conditions as the nutrients in the medium were going to be exhausted (König et al., 2010; Sun et al., 2017). According to the nitrogen balance in the aerobic nitrite denitrification process of strain YT03 (Table 1), 55.55% of the initial inorganic nitrogen (mainly nitrite nitrogen) was transferred to bacterial nitrogen for the growth of strain YT03, and TN loss reached 28.33%, which was degraded into gaseous products.

**TABLE 1:** Nitrogen balance during aerobic denitrification by YT03 with nitrite sole nitrogen source (mg L^–1^)^∗^.

**Forms of nitrogen**	**Inorganic nitrogen**			**Bacterial nitrogen**	**Nitrogen transferred to bacterial nitrogen (%)**	**Nitrogen removal rate (%)**
	**${\text{NH}}_{4}^{+}$-N**	**${\text{NO}}_{2}^{-}$-N**	**${\text{NO}}_{3}^{-}$-N**			
Initial	0.69 ± 0.10	9.27 ± 0.03	–	1.07 ± 0.15	–	–
Final	1.32 ± 0.05	–	–	6.53 ± 0.20	55.55 ± 1.78	28.33 ± 1.09

*^∗^Means undetected, and nitrogen ratio transferred to bacterial nitrogen and nitrogen removal rate were calculated by the formulas:*$\begin{array}{l} Nitrogen\;transferred\;to\;bacterial\;nitrogen\;(\%)=\frac{\left(final\;bacterial\;nitrogen-initial\;bacterial\;nitrogen\right)}{initial\;total\;inorganic\;nitrogen}\times 100\%.\\ Nitrogen\;removal\;rate\;(\%)\;=\frac{\left(initial\;total\;nitrogen-final\;total\;nitrogen\right)}{initial\;total\;nitrogen}\times 100\%. \end{array}$

The comparison of nitrite denitrification activities of different strains from reported studies and strain YT03 was shown in Table 2. The average nitrite denitrification rate of 1.67 mg L^–1^ h^–1^ by strain YT03 is higher than these by *Bacillus* sp. YX-6 (Song et al., 2011), *Pseudomonas* sp. yy7 (Wan et al., 2011), and *Bacillus cereus* PB88 (Barman et al., 2017), with less usage of carbon source than the latter. Although *Acinetobacter* sp. Y1 (Liu et al., 2015) and *Acinetobacter junii* YB (Ren et al., 2014) showed higher nitrite denitrification rate than strain YT03, the finally residual nitrite concentration did not seem to be decreased further by these strains with the prolongation of reaction time. Therefore, by comparison, strain YT03 is a relatively promising aerobic denitrifier for removing nitrite from water environments.

**TABLE 2:** Comparison of the denitrification activities of different strains.

**Bacteria**	**Initial nitrite nitrogen concentration (mg L^–1^)**	**OD_600_**	**C/N ratio**	**Denitrification rate (mg L^–1^ h^–1^)**	**Removal efficiency (%)**	**References**
*Bacillus* sp. YX-6	10	0.6–1.4	140	0.71	100	Song et al., 2011
*Pseudomonas* sp. yy7	18.3	41–176 mg/L (Cell density)	16.3	0.76	97.3	Wan et al., 2011
*Acinetobacter* sp. Y1	100	0–1.2	13.7	2.29	80.5	Liu et al., 2015
*Bacillus cereus* PB88	12	0–1.5 (OD_620_)	140	0.25	82.33	Barman et al., 2017
*Acinetobacter junii* YB	200	0–1.6	10	2.86	85.67	Ren et al., 2014
*Acinetobacter* sp. YT03	10	0.042–0.178 (or 1.07–6.53 mg/L, biomass-N)	16	1.67	100	This study

### Different Factors Affecting Nitrite Denitrification by Strain YT03

#### Carbon Source

**FIGURE 3:**
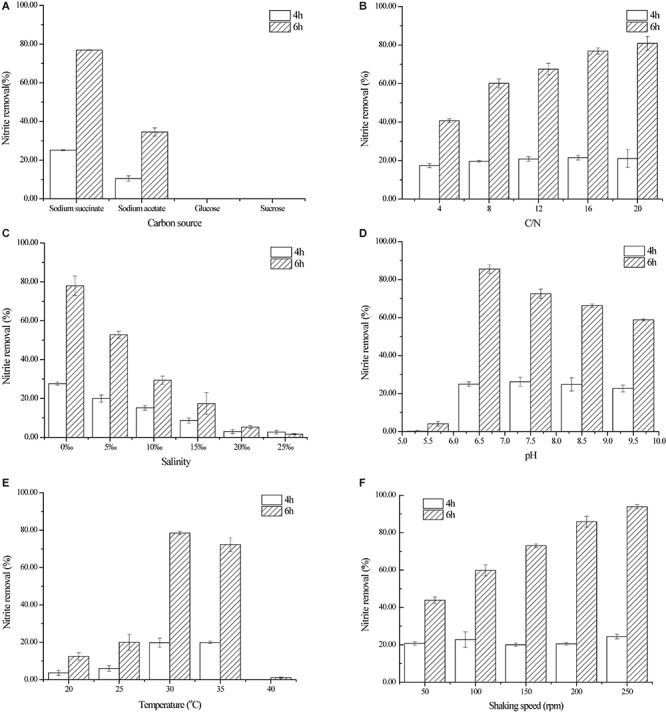
Effect of different factors on nitrite denitrification by strain YT03. **(A)** Carbon source, **(B)** C/N ratio, **(C)** salinity, **(D)** initial pH, **(E)** temperature, and **(F)** shaking speed. Error bars mean ±SD of three replicates.

The selection of carbon sources has an important impact on the behavior of heterotrophic denitrification bacterium in removal of nitrogen-containing pollutants. Four carbon sources were employed to investigate their roles in nitrite denitrification by strain YT03 in this study (Figure 3A). The concentration of nitrite was effectively decreased by strain YT03 after 6 h when sodium succinate was adopted as the sole carbon source, and the nitrogen removal efficiency reached the highest (76.96%) among the four kinds of tested carbon sources. While with sodium acetate as the carbon source, only 34.56% of nitrite was removed by strain YT03 in 6 h, which was much lower than that with sodium succinate. However, no nitrite decline was detected out with glucose or sucrose selected as the only carbon source, thus glucose or sucrose might not be a suitable carbon source for nitrite denitrification of strain YT03.

It was reported that the type of carbon source determined the complex biochemical pathways and enzymes involved in the denitrification process (Cherchi et al., 2009). Also, the carbon source with a simpler structure and smaller molecular weight, resulting in simpler biochemical pathways of carbon source utilization, would be more usable for denitrification bacteria (Cherchi et al., 2009; Zheng et al., 2011). In this study, sodium succinate and sodium acetate, with simpler structures and smaller molecular weights, resulted in higher nitrite removal efficiencies than glucose and sucrose. Similar conclusions were also drawn in aerobic denitrification of nitrate nitrogen by *Psychrobacter* sp. S1-1 (Zheng et al., 2011) and *Pseudomonas* sp. WSH1001 (Qiu et al., 2012). Therefore, in this study, sodium succinate was selected as the carbon source for aerobic nitrite denitrification by strain YT03, which could also be found in nitrite denitrification by *Bacillus* sp. YX-6 (Song et al., 2011), *B. cereus* PB88 (Barman et al., 2017), and *A. junii* YB (Ren et al., 2014).

#### C/N Ratio

Insufficient carbon source is unfavorable to the utilization and denitrification of nitrite by strain YT03, while excessive carbon source addition would be a waste and result in too high initial COD concentration (Zhao et al., 2018). Therefore, it is very necessary to investigate the optimal amount of carbon source added to the cultivation medium of strain YT03. As shown in Figure 3B, when insufficient carbon source (C/N = 4) was supplied for strain YT03, the nitrite removal rate was <50% (only 40.68%). After that, as the C/N ratio increased from 4 to 16, the nitrite removal rate increased significantly to 76.90%. However, the increasing of C/N ratio from 16 to 20 did not cause further significantly increasing of nitrite removal rate by strain YT03. Considering the cost and nitrite removal efficiency, 16 was chosen for the optimal C/N ratio for strain YT03.

Figure 3B demonstrated the nitrite denitrification efficiency kept increasing as with the improving of dosage of carbon source (C/N) among a certain C/N ratio range, which was consistent with the conclusion drawn from the nitrate denitrification of *Psychrobacter* sp. S1-1 (Zheng et al., 2011) and *Marinobacter* sp. F6 (Zheng et al., 2012), and ammonium removal of heterotrophic nitrifying-aerobic denitrifying bacteria including *A. junii* YB, *Pseudomonas putida* YH, and *Pseudomonas aeruginosa* YL (Yang et al., 2016). According to literatures, the optimal C/N ratios for aerobic denitrifiers *sychrobacter* sp. S1-1 (Zheng et al., 2011) and *Marinobacter* sp. F6 (Zheng et al., 2012) and *Bacillus methylotrophicus* L7 (Zhang et al., 2012) were 15, 15, and 20, respectively, which were also close to the optimal C/N ratio (16) in this work.

#### Salinity

Figure 3C shows the effect of salinity on nitrite denitrification by strain YT03. As seen from Figure 3C, the salinity also put a significant influence on the nitrite denitrification performance of YT03. The nitrite removal rate in 6 h by YT03 dropped significantly from 77.98 to 29.33% when the salinity in the cultivation medium increased from 0 to 10‰. And as the salinity kept rising to over 15‰, the nitrite removal rate decreased to far <20%, indicating that high salinity (over 15‰) of the medium would seriously inhibit the nitrite utilization and degradation of strain YT03.

Salinity may cause different influences on bacteria due to their origin. The strain YT03 was isolated from freshwater, and its denitrification activity decreased obviously with the increasing of salinity (from 0 to 25‰), and similar influences were observed in *B. methylotrophicus* L7 (Zhang et al., 2012) and *Bacillus* sp. YX-6 (Song et al., 2011). However, for marine origin aerobic nitrifying–denitrifying bacteria, *Psychrobacter* sp. S1-1 (Zheng et al., 2011), *Marinobacter* sp. F6 (Zheng et al., 2012), and *B. cereus* PB88 (Barman et al., 2017) displayed the highest aerobic denitrification activities at salinities of 10 g/L, 32–35 g/L, and 20‰, while at salinities lower or higher than the optimum values, their aerobic denitrification activities decreased. Therefore, the investigation of effects of salinity on denitrification by different strains is very necessary. And for strain YT03, the acclimation might need to be conducted first before it was applied to marine environment for nitrite removal.

#### Initial pH

Figure 3D shows the effect of initial pH on aerobic nitrite denitrification by strain YT03 with the initial pH range of 5.5–9.5. The nitrite removal rate was below 20% in 6 h when the initial pH of the culture medium was adjusted to 5.5. While the maximum nitrite removal rate 91.67% was reached at a little higher pH value of 6.5. However, as the initial pH increased from 6.5 to 9.5, the nitrite removal rate in 6 h dropped gradually to 49.21%. It could be concluded that the proper initial pH range for aerobic nitrite denitrification by strain YT03 was 6.5–8.5, among which the nitrite removal rate was kept at not <60%, while a pH of 6.5 was chosen as the optimum pH for aerobic nitrite denitrification by strain YT03. In addition, acidic conditions seemed unsuitable for the aerobic nitrite denitrification by strain YT03 as the nitrite removal rate was the lowest at initial pH of 5.5 among the experimental initial pH range, and also for other aerobic denitrifiers such low aerobic denitrification occurred at pH of 5.5 and 6.5 for *Marinobacter* sp. F6 (Zheng et al., 2012) and pH of 6 for *Pseudomonas stutzeri* PCN-1 (Zheng et al., 2014).

#### Temperature

The effect of temperature on aerobic nitrite denitrification by strain YT03 was shown in Figure 3E. At the cultivation temperatures of 20 and 25°C, the nitrite removal rate in 6 h was very low (no >20%). However, the nitrite removal rate witnessed a big rise to 78.48% at a slightly higher temperature of 30°C, and a slight drop of nitrite removal rate in 6 h occurred as the temperature increased from 30 to 35°C, while a sharp one occurred as the temperature continued increasing to 40°C. It was obvious that the optimal temperature for nitrite denitrification by strain YT03 was 30°C, which was inconsistent with other aerobic nitrite denitrifiers, such as *Bacillus* sp. YX-6 (Song et al., 2011) and *B. cereus* PB88 (Barman et al., 2017). Also, a temperature range of 30–35°C was proper for the aerobic nitrite denitrification for strain YT03, resulting in acceptable nitrite removal performance. Furthermore, it seemed that any temperature (i.e., 40°C) higher than 35°C or lower (i.e., 20 or 25°C) than 30°C was not suitable for nitrite denitrification by strain YT03. Temperature is one of the important factors affecting the catalytic efficiency of denitrifying enzymes, thus the nitrite reductase of aerobic denitrifier YT03 might be sensitive to temperature (Zhao et al., 2018).

#### Shaking Speed

Under aerobic cultivation conditions, higher shaking speed means higher dissolved oxygen, which is necessary for the high denitrification activity of aerobic nitrite denitrifiers (Song et al., 2011; Barman et al., 2017). As shown in Figure 3F, the nitrite removal rate kept increasing significantly from 43.86 to 93.85% with the shaking speed increasing from 50 to 250 rpm, which is consistent with the performance of *Bacillus* sp. YX-6 (Song et al., 2011) under similar shaking speed range (0–250 rpm). However, as the shaking speed rose, the aerobic nitrite denitrification efficiency of *B. cereus* PB88 (Barman et al., 2017) began declining after reaching a peak at 150 rpm, which was in contrast with the aerobic nitrite denitrification performance of strain YT03 in this study.

### Utilization of Ammonium by Strain YT03

**FIGURE 4:**
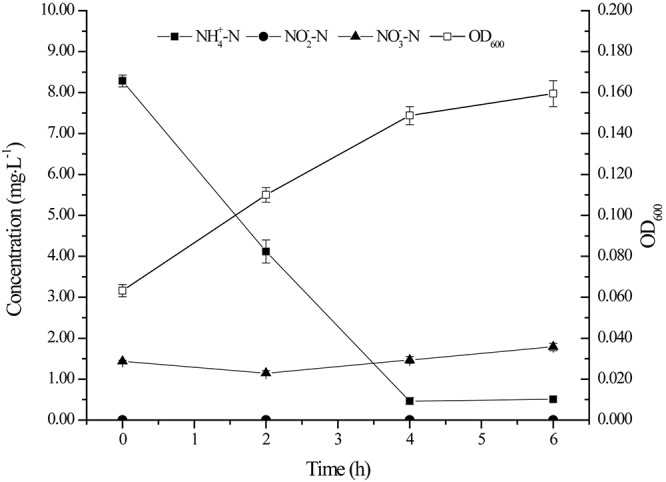
The growth and ammonium nitrogen removal by strain YT03. Error bars mean ±SD of three replicates.

Many aerobic denitrifying bacteria performed heterotrophic nitrification capacity (Zhang et al., 2012; Zheng et al., 2012; Zhou et al., 2014). Therefore, the ability of ammonium nitrogen utilization of the strain YT03 with ammonium chloride as nitrogen source was tested (Figure 4). As shown in Figure 4, YT03 demonstrated effective ammonium nitrogen ability and the ammonium nitrogen under an initial concentration of 8.28 mg L^–1^ was almost removed in 4 h, reaching an ammonium nitrogen removal efficiency of 94.41% and an average ammonium nitrogen heterotrophic nitrification rate of 1.96 mg L^–1^ h^–1^. OD_600_ increased from 0.063 to 0.160 during 6 h. There was no nitrite nitrogen accumulation during the ammonium nitrogen removal process; however, certain amount of nitrate nitrogen existed at the beginning of the process, which might be produced by the oxidation of ammonium nitrogen under natural condition, and did not seem to decline till the final stage of the process.

### Quality Assessment of RNA-Seq

**FIGURE 5:**
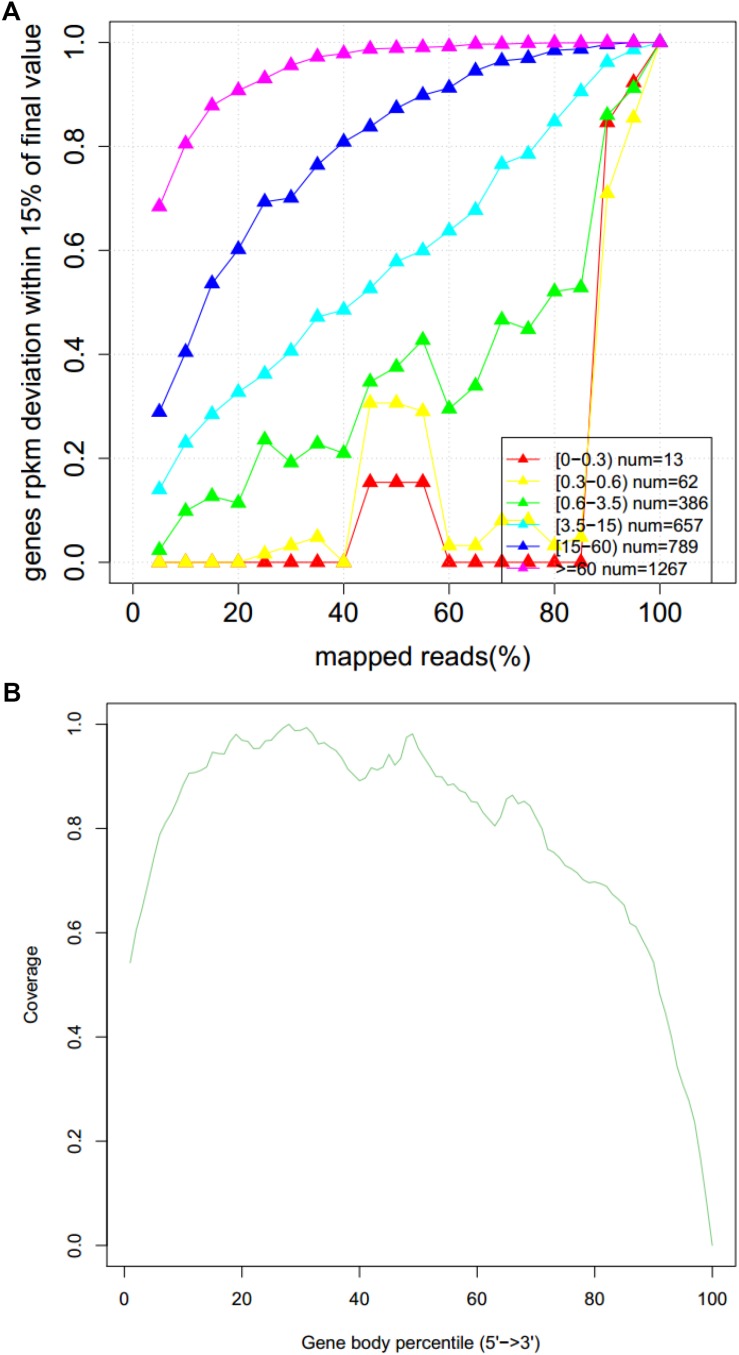
Sequencing saturation curve **(A)** and gene coverage analysis **(B)**.

The abscissa of Figure 5A represents the percentage of valid comparisons to reads, the overall quality of the saturation is high, and the sequence quantity can cover most of the expressed genes. The abscissa of Figure 5B is the percentage of the base length of a single gene to the total base length. There are no obvious peaks at the left and right ends of this map, which indicates that the sequencing results are not biased and the results are more uniform.

### Differentially Expressed Genes Analysis

**FIGURE 6:**
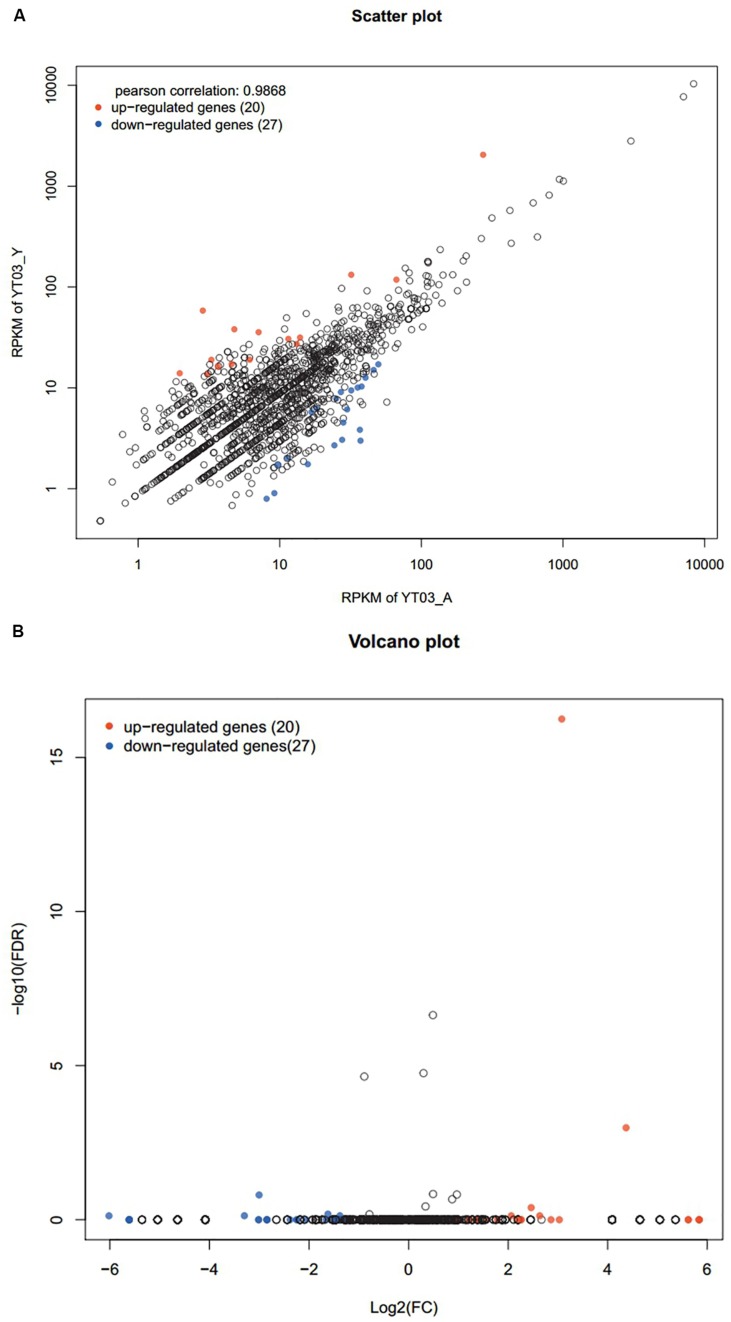
Scatter plot **(A)** and volcanic plot **(B)** of differentially expressed genes (red dots indicate significantly up-regulated genes, blue dots indicate significantly down-regulated genes, and black dots are non-significantly different genes).

**FIGURE 7:**
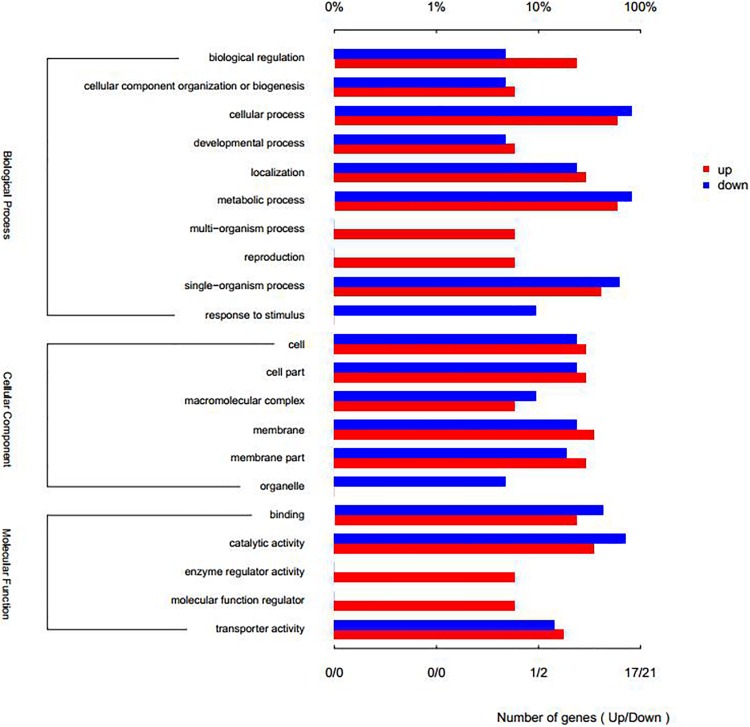
Column map of GO annotation for up-and-down differential genes.

Compared with the control group, there were 47 genes with significant differences in expression (*P* < 0.05), of which 20 genes were significantly up-regulated and 27 genes were significantly down-regulated (Figures 6A,B). Through GO annotation (Figure 7), it was found that mainly the up-regulated genes were involved in biological regulation, multi-organism process, reproduction, membrane, enzyme regulator activity, and molecular function regulator; and the down-regulated genes were related to cellular process, metabolic process, single-organism process, response to stimulus, macromolecular complex, organelle, binding, and catalytic activity.

**FIGURE 8:**
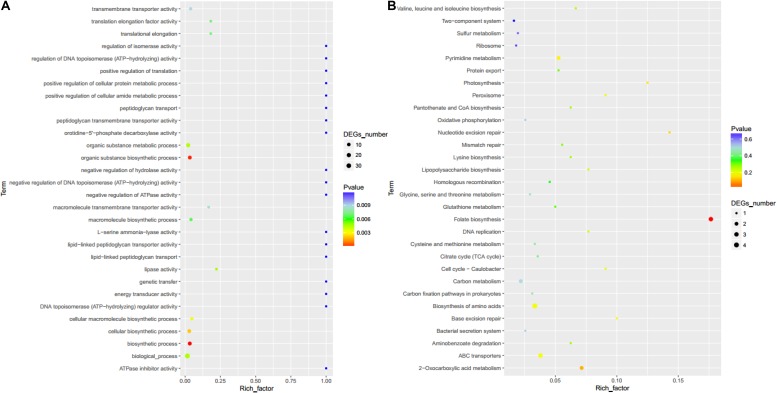
GO enrichment map **(A)** and KEGG enrichment map **(B)**.

Furthermore, GO enrichment analysis showed that differentially expressed genes were mainly concentrated in the organic substance metabolic process, organic substance biosynthetic process, biosynthetic process, biological process, and cellular biosynthetic process pathways (Figure 8A). KEGG enrichment analysis showed that differentially expressed genes were mainly concentrated in folate biosynthesis, biosynthesis of amino acids, ABC transporters, pyrimidine metabolism, and 2-oxocarboxylic acid metabolism pathways (Figure 8B).

### Key Genes Associated With Nitrogen Removal

Genes encoding ion channel protein (*Tsx*), C4-dicarboxylate transporter (*Dct*A), ABC transporter permease (ABC-2.P), twin-arginine translocase subunit (*Tat*B), elongation factor 4 (*Lep*A), and putative lipid II flippase (*Fts*W), which were involved in material transport of the bacterium, were up-regulated, possibly indicating the materials transport in strain YT03 for aerobic nitrite denitrification was promoted compared with that for ammonium nitrogen utilization under aerobic condition. Furthermore, C4-dicarboxylate transporter, encoded by the up-regulated gene *Dct*A, was reported to participate in the transport of succinate (Unden and Bongaerts, 1997), further contributing to the carbon metabolism in aerobic nitrite denitrification of YT03 with sodium succinate as carbon source.

Genes *Dap*A encoding dihydrodipicolinate synthase (DHDPS) family protein, *Pab*B encoding anthranilate synthase component I family protein were up-regulated, and were involved in the first committed synthesis step of dihydrodipicolinate and anthranilate synthesis, respectively. Involved in pyrimidine synthesis, genes *Pyr*C encoding dihydroorotase and PyrF encoding orotidine 5′-phosphate decarboxylase were also up-regulated (Truong et al., 2013; Ito et al., 2016). Therefore, these up-regulated genes and relevant enzymes and biosynthetic processes might be closely related to the strain YT03 for nitrite nitrogen utilization and need to be further investigated.

The use of nitrite either as substrate for nitrogen assimilation or as electron acceptor requires its active transport through the cytoplasmic membrane to reach the concentrations required for the assimilative or respiratory nitrite reductases to function (Wang et al., 2008; Alvarez et al., 2018). Gene *Nrt* encoding nitrate/nitrite transporter was up-regulated and might play an important role in the aerobic nitrite denitrification process of strain YT03.

## Conclusion

A novel aerobic denitrifier YT03 able to utilize nitrite as the solo nitrogen source was isolated and characterized in this study. Strain YT03 could completely remove 10 mg L^–1^ of nitrite from water in only 6 h under aerobic condition. The average nitrite removal rate was 1.67 mg L^–1^ h^–1^, which was more effective than most bacteria reported in some previous studies. The optimum culturing condition of strain YT03 was easy to satisfy. All this evidence indicates that strain YT03 is a very promising aerobic denitrifier for removing nitrite from water environment and addressing the nitrite pollution problems. The results of transcriptome analysis showed that the differentially expressed genes were mainly involved in transport process of carbon source and other nutrients; biosynthetic process of dihydrodipicolinate, anthranilate, and pyrimidine; and transport of nitrite for assimilative or respiratory nitrite reductases. Especially, the genes related to the transport of carbon source (*Dct*A) and denitrification substrate (*Nrt*) might demonstrate the importance of active substrate transport for efficient substrate utilization in aerobic nitrite denitrification with sodium succinate by strain YT03. Further investigation would be required to confirm the concrete relationship between the process related to differentially expressed genes and the aerobic nitrite removal by strain YT03, for better understanding of the mechanism of nitrite-based aerobic denitrification.

## Data Availability Statement

The raw data supporting the conclusions of this manuscript will be made available by the authors, without undue reservation, to any qualified researcher, upon reasonable request.

## Author Contributions

BL and RL made substantial contributions to the acquisition, and analysis and interpretation of data for the work, and drafted the work. YX, WH, YM, and JZ revised it critically for important intellectual content. LL and XH agreed to be accountable for all aspects of the work in ensuring that questions related to the accuracy or integrity of any part of the work that are appropriately investigated and resolved.

## Conflict of Interest

The authors declare that the research was conducted in the absence of any commercial or financial relationships that could be construed as a potential conflict of interest.

## Footnotes

1 http://www.geneontology.org/

2 https://github.com/tanghaibao/GOatools
